# Theasaponin E_1_ Inhibits Platinum-Resistant Ovarian Cancer Cells through Activating Apoptosis and Suppressing Angiogenesis

**DOI:** 10.3390/molecules26061681

**Published:** 2021-03-17

**Authors:** Bo Li, Tuantuan Tong, Ning Ren, Gary O. Rankin, Yon Rojanasakul, Youying Tu, Yi Charlie Chen

**Affiliations:** 1 Department of Tea Science, Zhejiang University, Hangzhou 310058, China; drlib@zju.edu.cn (B.L.); tongtuantuan@zju.edu.cn (T.T.); ningren@zju.edu.cn (N.R.); 2 College of Science, Technology and Mathematics, Alderson Broaddus University, Philippi, WV 26416, USA; 3 Department of Biomedical Sciences, Joan C. Edwards School of Medicine, Marshall University, Huntington, WV 25755, USA; rankin@marshall.edu; 4 Department of Pharmaceutical Sciences, West Virginia University, Morgantown, WV 26506, USA; yrojan@hsc.wvu.edu

**Keywords:** ovarian cancer, theasaponin E_1_, *Camellia sinensis* seeds, apoptosis, cell cycle arrest, angiogenesis

## Abstract

Novel therapeutic strategies for ovarian cancer treatment are in critical need due to the chemoresistance and adverse side effects of platinum-based chemotherapy. Theasaponin E_1_ (TSE1) is an oleanane-type saponin from *Camellia sinensis* seeds. Its apoptosis-inducing, cell cycle arresting and antiangiogenesis activities against platinum-resistant ovarian cancer cells were elucidated in vitro and using the chicken chorioallantoic membrane (CAM) assay. The results showed that TSE1 had more potent cell growth inhibitory effects on ovarian cancer OVCAR-3 and A2780/CP70 cells than cisplatin and was lower in cytotoxicity to normal ovarian IOSE-364 cells. TSE1 significantly induced OVCAR-3 cell apoptosis via the intrinsic and extrinsic apoptotic pathways, slightly arresting cell cycle at the G2/M phase, and obviously inhibited OVCAR-3 cell migration and angiogenesis with reducing the protein secretion and expression of vascular endothelial growth factor (VEGF). Western bolt assay showed that Serine/threonine Kinase (Akt) signaling related proteins including Ataxia telangiectasia mutated kinase (ATM), Phosphatase and tensin homolog (PTEN), Akt, Mammalian target of rapamycin (mTOR), Ribosome S6 protein kinase (p70S6K) and e IF4E-binding protein 1(4E-BP1) were regulated, and Hypoxia inducible factor-1α (HIF-1α) protein expression was decreased by TSE1 in OVCAR-3 cells. Moreover, TSE1 treatment potently downregulated protein expression of the Notch ligands including Delta-like protein 4 (Dll4) and Jagged1, and reduced the protein level of the intracellular domain (NICD) of Notch1. Combination treatment of TSE1 with the Notch1 signaling inhibitor *tert*-butyl (2*S*)-2-[[(2*S*)-2-[[2-(3,5-difluorophenyl)acetyl]amino]propanoyl]amino]-2-phenylacetate (DAPT), or the Akt signaling inhibitor wortmannin, showed a stronger inhibition toward HIF-1α activation compared with single compound treatment. Taken together, TSE1 might be a potential candidate compound for improving platinum-resistant ovarian cancer treatment via Dll4/Jagged1-Notch1-Akt-HIF-1α axis.

## 1. Introduction

Ovarian cancer is the most lethal malignancy of all female reproductive cancers, and ranks fifth in cancer deaths among women worldwide [1]. Its incidence rates vary by regions and countries due to differences in cancer histotype, risk factors and biomarkers [2]. Europe and North America are higher areas of ovarian cancer incidence compared with Asia and Africa [3]. In 2019, the new cases and deaths of ovarian cancer in the United States were estimated to be 22,530 and 14,240, respectively, with a five-year survival rate of 47.6%. The high mortality of ovarian cancer is mostly due to late diagnosis, resulting from the lack of specific clinical symptoms and effective screening methods, and because of a high recurrence rate [4]. Cytoreductive surgery combined with platinum-based chemotherapy is the first-line therapy [5]. However, acquired resistance to platinum is a major problem that leads to recurrence of disease and severely hinders long-term survival [6]. Most patients with advanced-stage ovarian cancer have a recurrence of the tumor within two years. Moreover, a lack of cancer cell selectivity by these drugs results in normal tissue toxicity [7]. Despite development of targeted therapies and improvements in chemotherapy, the patient survival rate has not notably improved in the recent years [8]. Novel alternative strategies and drugs with high therapeutic efficiency and limited side effects are in critical need.

Apoptosis-based and antiangiogenic therapies have become new research areas for the treatment of ovarian cancer within the last decade. Alteration of apoptosis pathways is an important hallmark of cancer cells, which allows them to evade the immune system and confer resistance to conventional chemotherapy, radiotherapy and biologic treatments [9]. The multicellular spheroids from ovarian cancer cells treated with cisplatin are more resistant to cell death compared with suspended and adherent ovarian cancer cells. Higher levels of antiapoptotic proteins, such as Bcl-2, and reduced Caspase-3 and -9 activities were determined in multicellular spheroids [10]. Caspase-3 and -8 levels in benign and malignant ovarian tumors are lower than in normal ovary tissues [11]. A high level of Caspase-8 correlates with increased overall survival of ovarian cancer patients [12,13].

Angiogenesis is crucial for ovarian cancer growth and metastasis in the peritoneal space. The vascular endothelial growth factor (VEGF) is an important regulator of angiogenesis [14]. VEGF-targeted drugs have been developed and clinically used for treating various types of solid tumors [15]. Combination treatment with angiogenesis inhibitors and conventional chemotherapy significantly improved the progression-free and overall survival in patients with a high-risk of progression and recurrent ovarian cancer [16]. Hypoxia is an important mechanism for cancer cells to evade antitumor immune responses. The tumor microenvironment is shaped under hypoxia, and hundreds of genes are regulated transcriptionally and post-translationally to modulate tumorigenesis, immune suppression, apoptosis, angiogenesis and drug-resistance. Hypoxia-inducible factor-1 (HIF-1) is a key transcription factor regulating the adaptive response to hypoxia. High HIF-1α expression is a prognostic factor in ovarian cancer [17]. HIF-1 α binds to the AEG-1 promoter to regulate ovarian cancer metastasis [18], and it induces autophagy associated with cisplatin resistance of ovarian cancer cells [19]. Targeting Hypoxia inducible factors (HIFs) or combining HIF inhibitors with other therapeutics could be an effective strategy to eradicate cancer stem cells and overcome cisplatin resistance in ovarian cancer [20].

Natural products have been important sources for the discovery of new drugs. Between 1999 and 2013, 28% of first-in-class drugs permitted by the Food and Drug Administration (FDA) were derived from natural pharmacophores. Currently, around 70% of anticancer drugs on the market are developed from natural products and their derivatives [21]. Saponins are a large group of secondary metabolites of plants including vegetables, fruits, tea, nuts and herbal medicines, and occur as triterpene or steroid glycosides. Dietary saponins have received more and more attention due to their potential nutritional effects [22]. These compounds have been shown to possess potent anticancer activity and have the capacity to reverse multidrug resistance (MDR) and reduce the side effects of radiotherapy and chemotherapy [23]. *Camellia sinensis* is famous worldwide for the popular beverage, tea, made from its leaves. In addition to the leaves, *C. sinensis* seeds are receiving more attention due to their various ingredients and bioactivities. Oleanane-type triterpene saponins are one of the main bioactive components of *C. sinensis* seeds and exert many pharmacological functions including antioxidant, anticancer, anti-inflammatory, antimicrobial, neuroprotective and hypolipidemic effects [24]. The total saponins of *C. sinensis* seeds significantly suppress ascites tumors in S180-implanted Institute of Cancer Research (ICR) mice when administered orally. Among seven saponin compounds (Theasaponin A_8_, A_9_, C_1_, E_l_, E_2_, H_1_ and assamsaponin C) from *C. sinensis* seeds, Theasaponin E_l_ exhibited the strongest proliferation inhibitory effects on promyelocytic leukemia cell line HL-60 and myelocytic leukemia K562 cells, and its activity was more potent than that of 4′-bromoflavone used as a positive control [25]. Recently, TSE1 was found to suppress the sphere forming and invasion activity of Aldehyde dehydrogenase (ALDH)-positive ovarian cancer stem-like cells (CSCs), which contribute to tumor growth, recurrence and chemoresistance [26]. However, its antiovarian cancer efficacy and underlying molecular mechanisms have not been clarified. In this work, we studied the effects of TSE1 on cell proliferation, apoptosis, cell cycle arrest and angiogenesis in the platinum-resistant ovarian cancer cells. The underlying signaling networks regulated by TSE1 in the OVCAR-3 cells were also explored.

## 2. Results

### 2.1. Cytotoxicity of TSE1 on OVCAR-3, A2780/CP70 and IOSE-364 Cells

**Figure 1:**
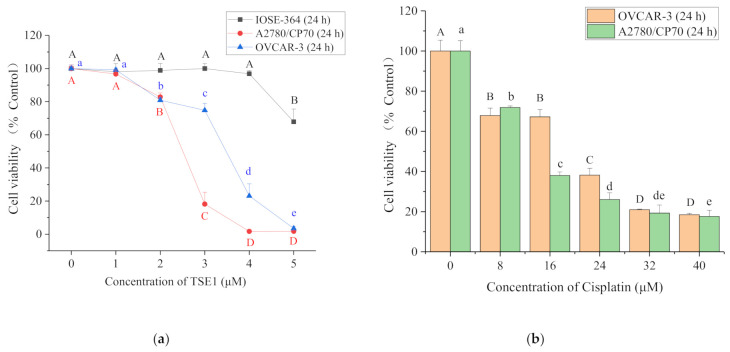
Effect of Theasaponin E_1_ (TSE1) and cisplatin on cell viability at 24 h. (**a**) TSE1 cytotoxicity effects in OVCAR-3, A2780/CP70 and IOSE-364 cells. (**b**) Cisplatin cytotoxicity in OVCAR-3 and A2780/CP70 cells. Cell viability was determined by the 3-(4,5-dimethylthiazol-2-yl)-5-(3-carboxymethonyphenol)-2-(4-sulfophenyl)-2*H*-tetrazolium (MTS) assay. Data represent means ± SD from three independent experiments. Significant differences among different treatments are indicated by different letters (*p* < 0.05).

To evaluate the cytotoxic effects of TSE1 and cisplatin on human ovarian cancer cell lines OVCAR-3 and A2780/CP70, as well as human normal epithelial ovarian cell line IOSE-364, cell viability was determined using the 3-(4,5-dimethylthiazol-2-yl)-5-(3-carboxymethonyphenol)-2-(4-sulfophenyl)-2*H*-tetrazolium (MTS) assay. As shown in Figure 1a, TSE1 significantly inhibited OVCAR-3 and A2780/CP70 cell growth in a concentration-dependent manner at 24 h (*p* < 0.05), with IC_50_ values of around 3.5 μM and 2.8 μM. The IC_50_ value of TSE1 for the IOSE-364 cells was more than 5 μM after 24 h treatment. TSE1 at 4 μM killed approximately 80% of OVCAR-3 cells and 98% of A2780/CP70 cells and had no obvious inhibitory effect on the IOSE-364 cells. The IC_50_ values of cisplatin against the OVCAR-3 and A2780/CP70 cells after 24 h treatments were estimated to be 21.0 μM and 13.1 μM, respectively (Figure 1b). These results suggested that TSE1 exhibited lower cytotoxicity to normal ovarian cells than to ovarian cancer cells, and its inhibitory activity on the platinum-resistant OVCAR-3 and A2780/CP70 cells was much stronger than cisplatin.

### 2.2. TSE1 Induced Apoptosis in OVCAR-3 Cells

**Figure 2:**
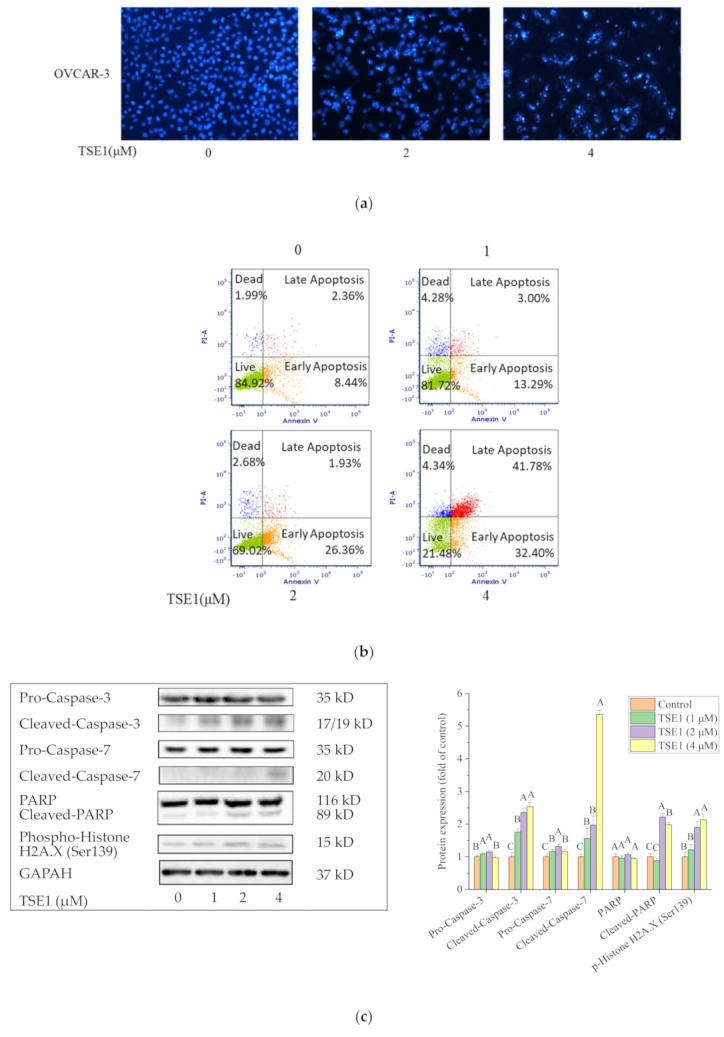

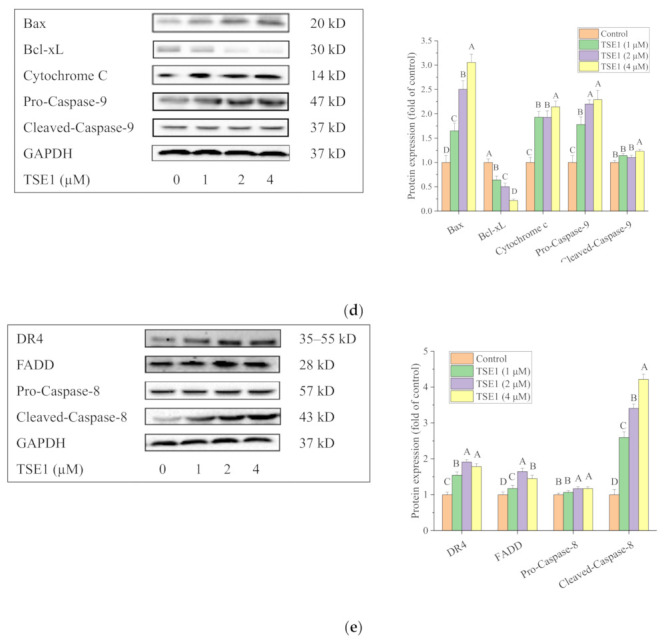
TSE1 induced apoptosis in OVCAR-3 cells after 24 h treatment. (**a**) Hoechst 33342 staining of OVCAR-3 detected by fluorescent microscopy (×400). (**b**) Flow cytometric analysis of OVCAR-3 cells using a double staining method with Fluorescein isothiocyanate (FITC)-conjugated Annexin V and PI. (**c**) Protein expressions of cell apoptosis and DNA damage markers including pro/cleaved-Caspase-3, pro/cleaved-Caspase-7, pro/cleaved-Poly (ADP-ribose) polymerase (PARP) andphospho-Histone H2A.X (Ser139). (**d**) Protein expressions of intrinsic apoptotic pathway-related proteins including Bax, Bcl-xL, Cytochrome C and pro/cleaved-Caspase-9. (**e**) Protein expressions of extrinsic apoptotic pathway-related proteins including DR4, FADD and pro/cleaved-Caspase-8. Protein expression were analyzed by Western blot. Results were expressed as mean ± SD from three independent experiments. Significant differences among different treatments are marked with different letters (*p* < 0.05).

Based on these initial studies, the OVCAR-3 cell line was used for further study. To determine whether TSE1 inhibits OVCAR-3 cell viability by inducing apoptosis, Hoechst 33342 DNA staining assay and flow cytometry were performed to analyze the cells treated with TSE1 (0–4 μM) for 24 h. Compared with the control group, brighter cells were observed after TSE1 treatment, indicating the occurrence of apoptotic cells with fragmented or condensed nuclei (Figure 2a). Flow cytometry analysis showed that the total percentage of early and late apoptotic cells increased from 10.80 to 74.18%, and the proportion of live cells decreased from 84.92 to 21.48% with TSE1 treatment in a concentration-dependent manner (Figure 2b). Moreover, the protein expression of cell apoptosis and DNA damage markers was determined by Western blot. TSE1 had no obvious effects on the protein expression of pro-Caspase-3, -7 (*p* > 0.05), and markedly increased the protein levels of cleaved-Caspase-3, cleaved-Caspase-7, cleaved-Poly (ADP-ribose) polymerase (PARP) and phospho-Histone H2A.X (Ser139) (*p* < 0.05), confirming that TSE1 induced apoptosis and DNA fragmentation in the OVCAR-3 cells (Figure 2c).

To clarify which apoptotic pathway is involved in TSE1-induced apoptosis, we determined the expression of key proteins in both signal transduction pathways. For the intrinsic apoptosis-regulating proteins, TSE1 significantly increased the expression of the proapoptotic protein Bax, Cytochrome C, pro- and cleaved-Caspase-9, and reduced the level of the antiapoptotic protein Bcl-xL (*p* < 0.05) (Figure 2d). With regard to the extrinsic apoptosis-related proteins, TSE1 upregulated the protein expression of Death receptor 4 (DR4), Fas-associating protein with a novel death domain (FADD), cleaved-Caspase-8 and slightly increased the pro-Caspase-8 protein level (*p* < 0.05) (Figure 2e). These results suggested that TSE1 induced apoptosis through both the intrinsic and extrinsic pathways in the OVCAR-3 cells.

### 2.3. TSE1 Induces G2/M Cell Cycle Arrest in OVCAR-3 Cell

**Figure 3:**
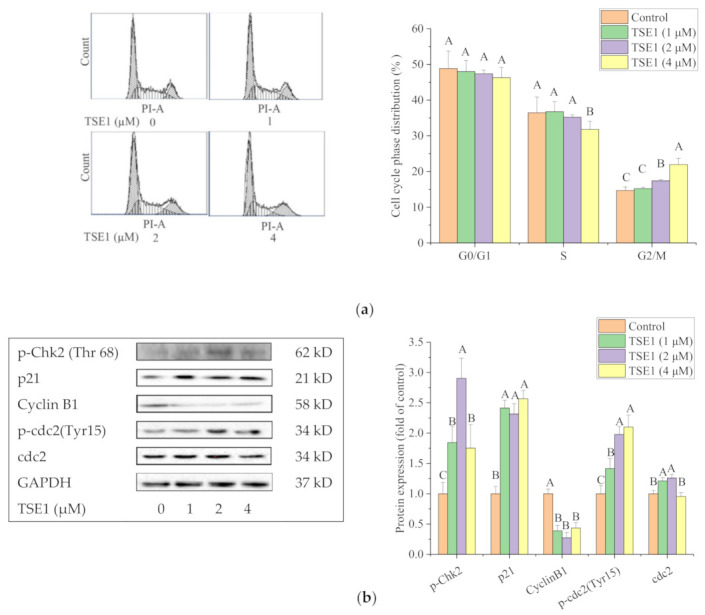
TSE1 induced cell cycle arrest at G2/M phase in OVCAR-3 cells after 24 h treatment. (**a**) Flow cytometry analysis of cell cycle phase distributions in OVCAR-3 cells. (**b**) Expression of G2/M cycle-related proteins including p-Chk2, p21, Cyclin B1, p-cdc2 and cdc2. Protein expression were analyzed by Western blot. Results were expressed as mean ± SD from three independent experiments. Significant differences among different treatments are marked with different letters (*p* < 0.05).

To make clear the role of cell cycle effects in TSE1-induced cell growth inhibition and apoptosis, the cell cycle phase distribution of TSE1-treated OVCAR-3 cells was analyzed by flow cytometry. The population of cells in the G2/M phase increased from 14.70 to 21.96% after exposure to TSE1 (0–4 µM) for 24 h, indicating that TSE1 slightly caused G2/M cell cycle arrest in OVCAR-3 cells (Figure 3a). Next, we determined the expression of proteins associated with G2/M cell cycle by Western blot. Figure 3b shows that TSE1 significantly increased the protein levels of p-Chk2, p21 and p-cdc2 (Tyr15), and downregulated Cyclin B1 protein expression (*p* < 0.05), suggesting that these proteins involved in the G2/M cell cycle arrest induced by TSE1.

### 2.4. TSE1 Inhibited the Migration and Angiogenesis

**Figure 4:**
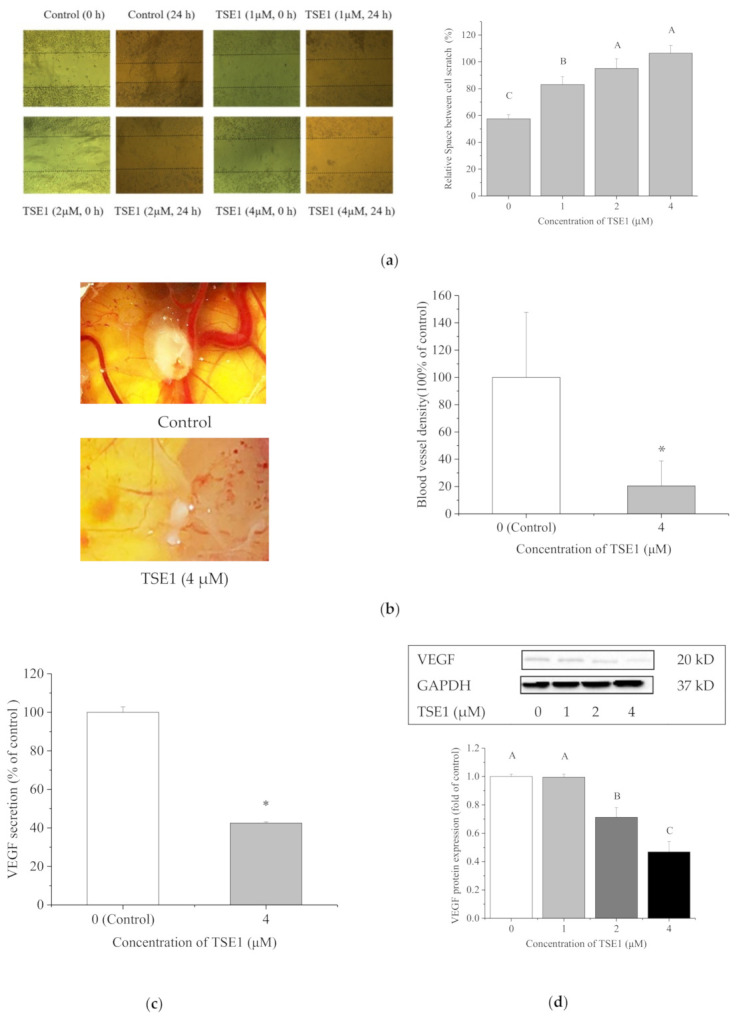
TSE1 inhibited OVCAR-3 cell migration and angiogenesis. (**a**) TSE1 suppressed the migration ability of OVCAR-3 cells determined by a wound healing assay after 24 h treatment. (**b**) TSE1 inhibited OVCAR-3 cell-induced blood vessel development in the chicken chorioallantoic membrane (CAM) model. (**c**) TSE1 inhibited VEGF secretion in OVCAR-3 cells analyzed by ELISA. (**d**) TSE1 reduced the VEGF protein level in OVCAR-3 cells detected by Western blot. Results were expressed as mean ± SD from three independent experiments. Significant differences among different treatments are marked with different letters (*p* < 0.05). * *p* < 0.05 versus control.

A wound healing assay was performed to investigate whether TSE1 repressed migration of the OVCAR-3 cells. As shown in Figure 4a, the relative space between the scratch of cells with TSE1 treatment (0–4 μM) increased from 57.50 to 100.00% at 24 h (*p* < 0.05), indicating TSE1 significantly inhibited the migration of OVCAR-3 cells. Cell migration is an important event that occurs during angiogenesis. Next, we evaluated the antiangiogenic activity of TSE1 in vivo using the chicken chorioallantoic membrane (CAM) assay. Highly vascularized structures were observed in the control group. When treated with 4 μM of TSE1, the number and density of CAM blood vessels were markedly reduced (Figure 4b).

Vascular endothelial growth factor (VEGF) is critical for tumor vascular development and maintenance. To investigate the effect of TSE1 on VEGF, its secretion in the cell culture and intracellular protein expression were determined by ELISA and Western blot, respectively. TSE1 at 4 μM reduced VEGF secretion by 57.5% compared with the control (Figure 4c). The protein level of VEGF in the cells was decreased by TSE1 (0–4 μM) in a concentration-dependent manner (Figure 4d). These results suggested that TSE1 inhibited OVCAR-3 cell induced-angiogenesis via targeting VEGF.

### 2.5. Effect of TSE1 on ATM/PTEN/Akt/mTOR/HIF-1α Pathway

**Figure 5:**
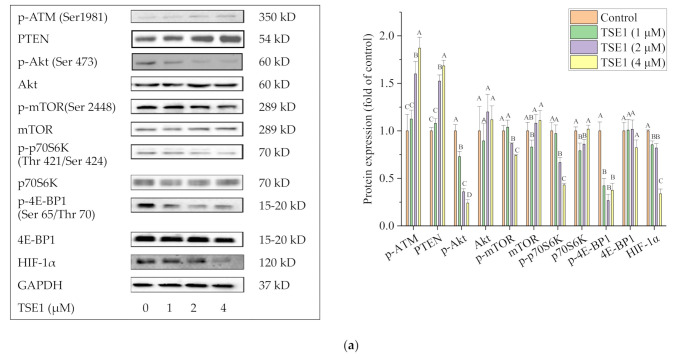

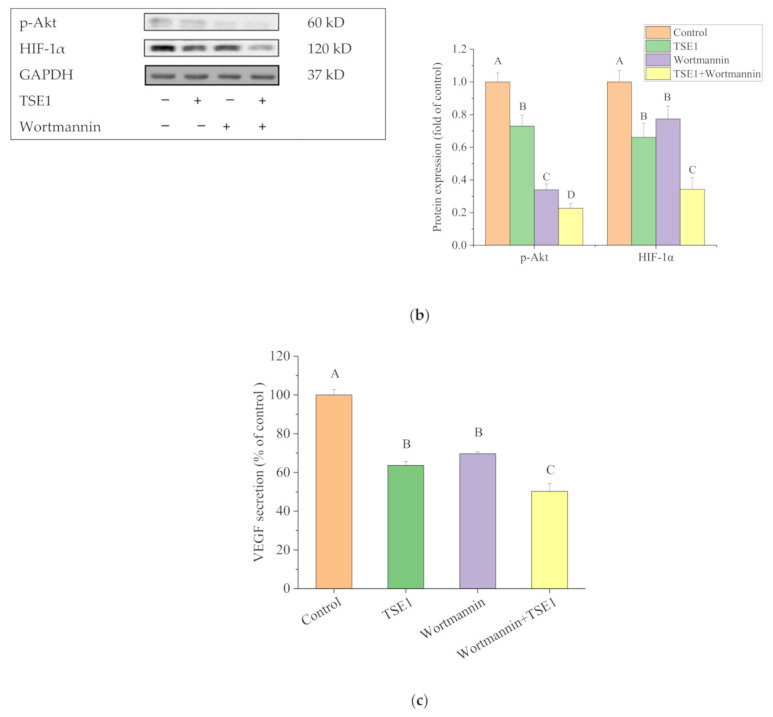
TSE1 regulated the ATM/PTEN/Akt/mTOR/HIF-1α pathway in OVCAR-3 cells after 24 h treatment. (**a**) Protein expressions of p-ATM, PTEN, p-Akt /Akt, p-mTOR/mTOR, p-p70S6K/p70S6K and p-4E-BP1/4EBP1. (**b**) Combination of TSE1 and wortmannin enhanced the suppressive effect on the protein expressions of p-Akt and HIF-1α compared with each single compound treatment. (**c**) Combination of TSE1 and wortmannin enhanced the inhibitory effect on VEGF secretion compared with each single compound treatment. Cells were treated with 2 µM of TSE1, 100 nM of wortmannin or their combination for 24 h. Protein expression was determined by Western blot, and VEGF secretion was determined by ELISA. Results were from three independent experiments and were expressed as means ± SD. Significant differences among different treatments are indicated by different letters (*p* < 0.05).

The effects of TSE1 on ATM, Akt signaling pathways and HIF-1α were further investigated. Figure 5a shows that TSE1 significantly increased the phosphorylation of ATM at Ser1981 and the protein expression of Phosphatase and tensin homolog (PTEN) decreased the phosphorylation of Akt (Ser 473) and its downstream proteins including mTOR (Ser 2448), p70S6K (Thr 421/Ser 424) and 4E-BP1 (Ser 65/Thr 70), and downregulated HIF-1α protein expression. When the cells were treated with the selective PI3K/Akt inhibitor wortmannin (100 nM), Akt phosphorylation, HIF-1α protein expression and VEGF secretion were significantly reduced, indicating that Akt regulated the expression of HIF-1α and VEGF in the OVCAR-3 cells. When the cells were cotreated by TSE1 (2 µM) and wortmannin (100 nM), the protein levels of p-Akt, HIF-1α and VEGF were more reduced compared with cells treated with TSE1 or wortmannin alone (Figure 5b,c). These results suggested that the ATM/Akt/HIF-1α axis might play an important role in the anticancer effects of TSE1. Wortmannin enhanced inhibitory effects of TSE1 on the Akt pathway and downstream effectors including VEGF and HIF-1α.

### 2.6. Effect of TSE1 on Dll4 and Jagged1-Mediated Notch 1 Signaling

**Figure 6:**
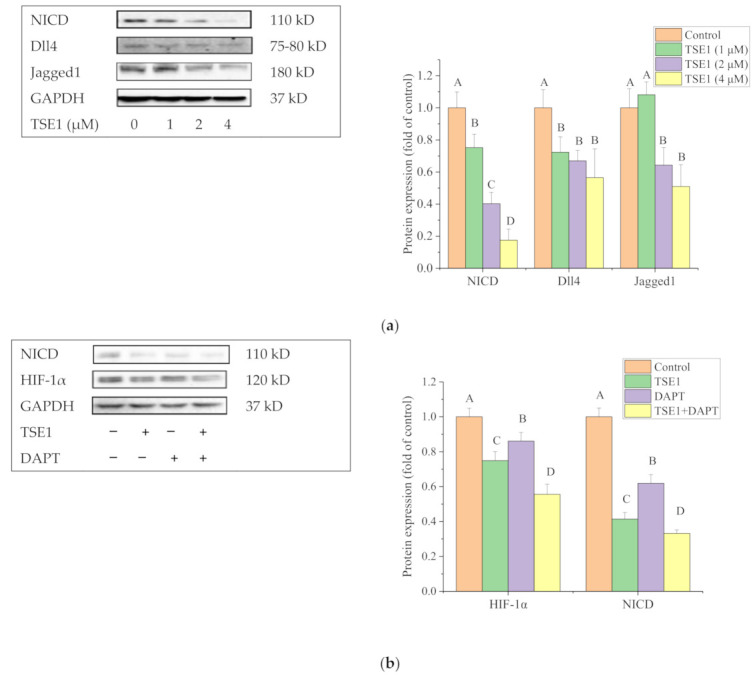
TSE1 regulated Notch1 pathway in OVCAR-3 cells after 24 h treatment. (**a**) Protein expressions of NICD, Dll4 and Jagged1. (**b**) Combination of TSE1 and DAPT enhanced the inhibitory effect on HIF-1α protein expression. Cells were treated with 2 µM of TSE1, 80 µM of DAPT or their combination for 24 h. Protein expression was determined by Western blot. Results were from three independent experiments and were expressed as means ± SD. Significant differences among different treatments are indicated by different letters (*p* < 0.05).

To investigate whether the Notch signaling pathway participated in the inhibitory effect of TSE1 in OVCAR-3 cells, we determined the released intracellular domain (NICD) of Notch1 and the ligands of the Notch receptor, including Delta-like protein 4 (Dll4) and Jagged1, by Western blot. Figure 6a shows that TSE1 significantly decreased the protein expression of NICD, Dll4 and Jagged1, indicating that TSE1 inhibited Notch 1 signal transduction, at least partly, through downregulating the Notch ligands and their following interaction with receptors. To clarify whether Notch 1 signaling could regulate HIF-1α in OVCAR-3 cells, the gamma-secretase inhibitor DAPT was used to treat the cells, which reduced the HIF-1α protein level. Moreover, NICD and HIF-1α protein expression in cells with DAPT and TSE1 cotreatment were lower than in cells treated with DAPT or TSE1 alone (Figure 6b). These results suggested that TSE1 regulated HIF-1α through the Notch 1 pathway, and this activity was enhanced in combination with DAPT.

## 3. Discussion

Platinum-based drugs, such as cisplatin, are important clinical drugs for the treatment of ovarian cancer. However, the high frequency of chemoresistance is a major challenge for improving the quality of life and overall survival in ovarian cancer patients. Moreover, the severe adverse side effects, including neurotoxicity, nephrotoxicity, cardiotoxicity and ototoxicity induced by platinum-based drugs have limited their broad clinical use. Complementary/alternative medicines are urgently needed for ovarian cancer therapy [27]. Although *C. sinensis* seed saponins have received increased attention as promising anticancer agents, the study of their pharmacology is still limited [24]. In this work, Theasaponin E_1_, one of the major saponins in the *C. sinensis* seeds, was proved to possess a much stronger proliferation-inhibitory effect than cisplatin in the cisplatin-resistant ovarian cancer cell lines OVCAR-3 and A2780/CP70. However, TSE1 induced lower cytotoxicity in normal ovarian IOSE-364 cells than in ovarian cancer cell lines (Figure 1).

Apoptosis is a process of programmed cell death, which maintains tissue homeostasis via eradicating damaged cells. Evasion from apoptosis is an important characteristic of tumor cells for survival, and causes acquired resistance to conventional anticancer therapeutics. Therefore, activation of apoptosis has been a major strategy for developing novel cancer therapies [28]. There are two classical signaling pathways of apoptosis: the intrinsic (mitochondrial-mediated) pathway and the extrinsic (death receptor-mediated) pathway. Intrinsic apoptosis is initiated by factors such as DNA damage, oxidative stress and nutrient deprivation. It is primarily regulated by the B-cell lymphoma 2 (Bcl-2) family proteins, which include pro and antiapoptotic members. The interactions between these proteins lead to mitochondrial outer membrane permeabilization. Subsequently, cytochrome c releases from mitochondria to the cytoplasm and binds with apoptotic peptidase activating factor 1 (Apaf-1) adaptor protein, Caspase-9 and dATP to form an apoptosome. This results in the activation of Caspase-3, poly ADP-ribose polymerase (PARP) cleavage and finally cell death [29]. In the past two decades, many natural and synthetic small molecule inhibitors of Bcl-2 family proteins have been developed for cancer treatment. Notably, some of these agents have entered clinical trials, and one compound, ABT-199, was approved by the U.S. Food and Drug Administration (FDA) in 2016 for treating chronic lymphocytic leukemia (CLL) [30]. The extrinsic apoptosis pathway is triggered by the binding of death ligands such as Fas ligand (FasL), tumor necrosis factor (TNF), and TNF-related apoptosis inducing ligand (TRAIL) to the death receptors (DRs). These DRs include TNFR1, FAS receptor (FASR), and DR 4/5 on the cell surface. This binding leads to the recruitment of the cytosolic death domain (DD)-containing adaptor proteins, such as Fas-associated protein with DD (FADD) on the cell surface. FADD contains an N-terminal death effector domain (DED) that can assemble with the activated receptor and pro Caspase-8 to from the death-inducing signaling complex (DISC). The assembly activates Caspase-8 and -3/7, followed by ultimately leading to apoptosis [31]. In this study, our results showed that TSE1 significantly induced apoptosis in OVCAR-3 cells via both the intrinsic and extrinsic pathways, by regulating the Bcl-2 family members (Bax and Bcl-xL), cytochrome C, DR4, FADD and the caspase cascade (caspase-8, -9 and 3/7) (Figure 2). Several oleanane-type triterpene saponins, such as Afrocyclamin A, Oleiferasaponin C-6 and Phaseoloideside E, have been reported to induce apoptosis in human prostate cancer cells, esophageal cancer cells, promyelocytic cells, hepatoma cells and gastric cancer cells though the intrinsic pathway [32,33,34]. Apoptosis induction may be one of the major molecular mechanisms of cancer cell death caused by these saponins. In addition, the phospho-Histone H2A.X (Ser139) protein level was significantly increased by TSE1 (Figure 2c). H2A.X is a core component of the nucleosome, and its phosphorylation on serine 139 (γH2A.X) is the major signal of DNA damage, which could trigger apoptosis through numerous factors and pathways including the intrinsic and extrinsic apoptosis pathways [35]. DNA damaging agents, such as methylating genotoxic agents, cisplatin and ionizing radiation play, an important role in cancer therapy [36]. Previous studies showed that Escin, a triterpene saponin from horse chestnut seeds, induced apoptosis in human colorectal cancer cells via p62/ATM/γH2A.X-mediated DNA damage [37]. These reports and our results suggested that DNA damage might partly contribute to TSE1-induced apoptosis in OVCAR-3 cells.

Cancer is a disease of inappropriate cell proliferation, which is controlled by cell cycle machinery. If cells fail to enter the next cell cycle stage they encounter transient arrest to repair DNA and then return to the cell cycle or move toward apoptosis after defective repair. Various classic chemotherapeutic agents exert their anticancer properties by disturbing the cell cycle [38]. Chk2 phosphorylation on Thr68 is believed to be responsible for G2/M arrest in a number of cancer cells. The protein p21WAF1, one of the downstream proteins of Chk2, regulates the formation of Cdc2/cyclin B1 complexes and G2/M transition [39,40]. Our cell cycle analysis showed that TSE1 induced G2/M cell cycle arrest in OVCAR-3 cells, at least in part, by regulating the phosphorylation of Chk2 and cdc2, and the protein expression of p21 and cyclin B1 (Figure 3). However, the cell percentage in the G2/M phase increased by approximate 10% after 4 μM TSE1 treatment for 24 h (Figure 3a), while the cell mortality and apoptosis rates were around 70–80% with the same treatment (Figure 1a and Figure 2b). This result suggested that the G2/M cell cycle arrest was only a minor mechanism of TSE1-induced OVCAR-3 cell growth inhibition and apoptosis. An oleanane-type saponin oleiferasaponin C6 from *Camellia oleifera* seeds was found to induce cell cycle arrest at the G0/G1 phases through regulating p21, cyclin-dependent kinase 4 (CDK4) and cyclin D1 in human promyelocytic leukemia HL-60 cells and hepatocellular carcinoma BEL-7402 cells. The percentage of the G0/G1 fraction was increased by 15–20% within the test dose [40]. This report, and our data, indicate that oleanane-type saponins with diverse chemical structures might have different effects on the cancer cell cycle.

Gynecological neoplasms, including ovarian cancer, are characterized by exacerbated angiogenesis, which is the process of new blood vessel generation. Angiogenesis plays a key role in supplying nutrients and oxygen to tumor tissues [41]. Antiangiogenic therapy is effective for many ovarian cancer patients who have failed conventional chemotherapy with low hematologic toxicity. VEGF is the principal and the most studied growth factor of tumor angiogenesis [42]. Although the antiangiogenic properties and VEGF inhibition by several tetracyclic triterpene saponins from ginseng, *Rhizoma paridis*, *Acanthopanax sessiliflorus* etc. have been reported, the knowledge about the effect of pentacyclic triterpene saponins on cancer angiogenesis is still limited [43,44,45,46]. In the present study, the wound healing assay showed that TSE1 almost totally inhibited OVCAR-3 cell migration at 2 μM after 24 h treatment (Figure 4a). It was noticed that cell growth inhibition and apoptosis rate with the same treatment were between 20 and 30%, indicating that the effective dose of TSE1 for inhibiting OVCAR-3 cell migration was lower than that for inducing cell death and apoptosis. The CAM assay showed that TSE1 obviously suppressed OVCAR-3 cell-induced angiogenesis in vivo (Figure 4b). In addition, TSE1 significantly reduced the extracellular secretion and intracellular protein expression of VEGF in OVCAR-3 cells (Figure 4c,d). Currently, available drugs approved by Food and Drug Administration (FDA) are targeting either VEGF or its receptor [47]. The effective inhibition of angiogenesis and VEGF by TSE1 highlights the potential of this compound for use as an antiangiogenic agent.

Tissue hypoxia is a key driver for angiogenic response and tumor progression [48]. The transcription factor HIF-1α regulates more than 100 genes involved in cancer cell proliferation, apoptosis and angiogenesis, and plays a key role in hypoxia-induced chemoresistance in ovarian cancers. Targeting HIF-1α is considered a promising approach to improve ovarian cancer outcomes [20]. The PI3K/Akt/mTOR signaling pathway is crucial in cell survival, proliferation, metabolism, nutrition regulation and tumorigenic processes [49]. The mTOR kinase exists in two different kinase complexes, mTORC1 and mTORC2. mTORC1 regulates HIF-1α translation protein through phosphorylating its downstream targets p70S6K and 4E-BP1 [50]. Our data showed that TSE1 significantly decreased HIF-1α protein expression, parallel with inhibiting the phosphorylation of Akt, mTOR, p70S6K and 4E-BP1 (Figure 5a). This result is consistent with the previous report that minocycline attenuates HIF-1α expression through the Akt/mTOR/p70S6K/4E-BP1 pathway in human ovarian carcinoma cell lines (A2780, OVCAR-3 and SKOV-3) and mice harboring OVCAR-3 xenografts [51]. However, whether or not HIF-1α locates downstream in the Akt pathway is still controversial. Huang et al. found that HIF-1α knockdown inhibited the PI3K/Akt/mTOR signaling pathway in A2780 and SKOV3 ovarian cancer cells [52]. To clarify this question, wortmannin, a PI3K inhibitor was used to treat the cells in this study. The protein level of HIF-1α was synchronously decreased with the reduced phosphorylation of Akt, indicating that HIF-1α was regulated by the PI3K/Akt signaling in the OVCAR-3 cells. Interestingly, the combination of TSE1 and wortmannin significantly enhanced the inhibitory effect on the Akt phosphorylation, HIF-1α protein expression and VEGF secretion compared with either individual compound treatment (Figure 5b,c). Wortmannin has been reported to synergistically enhance chemoradiotherapy with cisplatin or X-rays and reverse platinum and radio resistance in ovarian and bladder cancer models [53,54]. Our results indicated that the combination of TSE1 and wortmannin might be effective therapies for ovarian cancer.

Ataxia telangiectasia mutated kinase (ATM) belongs to the PIKK (PI3K-like protein kinases) family of serine/threonine kinases. Activation of ATM by autophosphorylation on Ser1981 regulates the cell-cycle checkpoint and DNA damage response following double strand breaks (DSBs) [55]. Abnormal expression of the ATM gene was reported to closely associate with poor prognosis in ovarian cancer patients. ATM targeting is considered as a promising anticancer strategy and has the potential to improve chemotherapy [56]. It was reported that ATM mediated the Akt/GSK-3β/Snail signaling pathway in ovarian cancer cells (A2780, Hey, HeyA8, SKOV3, SKOV3ip1, OVCA433 and OVCA429 cell lines), xenograft mouse models of ovarian cancer and high grade serous ovarian cancer patients [57,58]. In addition, ATM regulates PTEN, the antagonist of PI3K through p85α and XIAP mediated proteasome degradation in ovarian cancer cells (OVCAR3, OVCAR4) and human epithelial ovarian tumors [59]. Previous research showed that Gankyrin regulated HIF-1α protein stability via Akt activation by inhibiting PTEN in the Hey, HO8910 and ES2 ovarian cancer cell lines [60]. In the present work, the protein levels of p-ATM (Ser1981) and PTEN were enhanced after TSE1 treatment (Figure 5a), indicating that TSE1 might regulate the Akt/mTOR/p70S6K/4E-BP1/HIF1α pathway at least partly through the ATM/PTEN axis in OVCAR-3 cells. Moreover, the phosphorylation of H2A.X and activation of checkpoint kinase Chk2 are known to be regulated by ATM [61]. The increase of p-H2A.X (Ser139) and p-Chk2 (Thr 68) protein levels observed in this study (Figure 2c and Figure 3b) indicated that ATM might be associated with TSE1-induced DNA damage and cell cycle arrest.

**Figure 7:**
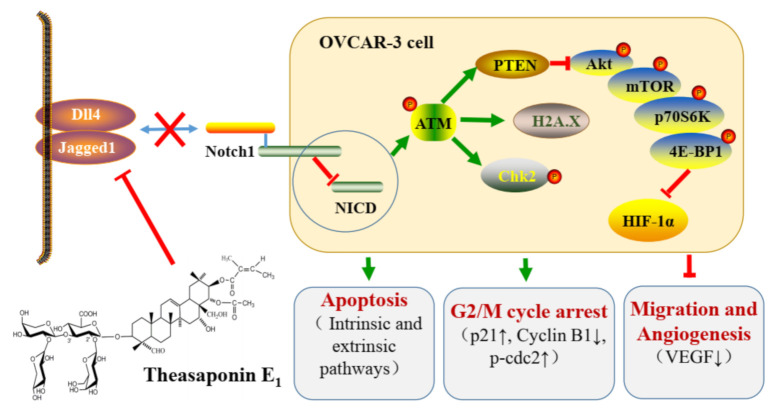
Schematic summary of the anticancer effect exerted by TSE1 on OVCAR-3 human ovarian cancer cells.

The evolutionary conserved Notch signaling pathway functions through cell-to-cell contact, and plays an established role in numerous developmental and physiological processes. Four Notch receptors (Notch1-4) and five ligands (Dll1, 3, 4 and Jagged 1, 2) have been identified in mammals. Notch receptors are activated after three cleavages carried out by furin-like proteases (S1), disintegrin and metalloprotease (ADAM) proteases (S2) and γ-secretase (S3), allowing the Notch intracellular domain (NICD) to translocate to the nucleus and form a transcriptional coregulator [62]. Growing evidence has suggested that Notch1 is important for tumor progression, chemoresistance and cancer stem-cell biology in ovarian cancer [63]. NICD-1 is overexpressed in ovarian cancer cells, and its depletion causes growth reduction [64]. The Notch ligands Dll4 and Jagged 1 play key roles in the ovarian cancer resistance to anti-VEGF therapy and platinum [65,66]. Our data showed that TSE1 significantly decreased the protein levels of NICD, Dll4 and Jagged1 (Figure 6a), indicating that this compound might inhibit Notch signaling, at least partly, through regulating the interaction between the Notch receptor and ligands and following S2 cleavage. It was reported that Notch 1 and ATM activation were inversely correlated in *Caenorhabditis elegans, Xenopus laevis* and humans. Notch 1 binds directly to the regulatory FRAP, ATM, TRRAP C-terminal (FATC) domain of ATM and inactivates ATM kinase [67]. Several recent studies have demonstrated that Notch 1 regulates PTEN/Akt signaling in ovarian cancer cells, hepatocytes and skeletal muscle cells [68,69,70]. These studies led us to explore whether the Notch 1 pathway regulates HIF-1α in OVCAR-3 cells, and the data showed that the Notch signaling inhibitor DAPT reduced HIF-1α protein expression (Figure 6b). These studies indicated that Notch signaling might be crucial for the regulation of the ATM/PTEN/Akt/HIF-1α pathway by TSE1 in OVCAR-3 cells. The proposed model of anticancer effect exerted by TSE1 on OVCAR-3 cells is shown in Figure 7. In addition, TSE1 was found to synergistically decrease the protein levels of NICD and HIF-1α together with DAPT (Figure 6b). The inhibitors of Notch signaling have been shown to strengthen the anticancer activity of other agents. Li et al. reported that Notch 1 inhibition by DAPT enhanced DNA damage and the sensitivity of cervical cancer cells to cisplatin [71]. Sequential combination of cisplatin prior to MK-0752, another inhibitor of Notch signaling, significantly induced cell apoptosis and suppressed the growth of ovarian cancer xenografts in nude mice [72]. Our results demonstrated that the combination of TSE1 and cisplatin might provide a more effective therapeutic strategy than cisplatin-only treatment for ovarian cancer. Saponins might enhance the uptake of other agents into cells via disrupting the membrane, and therefore increase compounds’ bioactivities [73]. The combined effect and mechanism of TSE1 with DAPT and wortmannin on ovarian cancer cells deserves further studies in the future.

## 4. Materials and Methods

### 4.1. Cell Culture and Reagents

The platinum-resistant human ovarian cancer cells (OVCAR-3 and A2780/CP70) were gifts from Dr. Bing-Hua Jiang from West Virginia University (Morgantown, WV, USA). The IOSE-364 normal ovarian surface epithelial cells were kindly provided by Dr. Nelly Ausperg from University of British Columbia (Vancouver, BC, Canada). Cells were maintained in RPMI-1640 medium (Sigma, St. Louis, MO, USA) containing 10% fetal bovine serum (FBS) at 37 °C with 5% CO_2_. TSE1 was prepared and identified as reported in our previous work [74].

### 4.2. Cell Viability Assay

Cell growth inhibition was determined using the MTS-based Cell Titer 96^®^Aqueous One Solution Cell Proliferation Assay kit (Promega, Madison, WI, USA). Cells were seeded into 96-well plates, incubated overnight, and then treated with TSE1 (0–5 μM) or cisplatin (0–40 μM) for 24 h. An equal amount of DMSO was used for control cells. After treatment, 20 µL of MTS solution was added to each well, and then incubated at 37 °C in the dark for one hour. The absorbance at 490 nm was measured using a Synergy™ HT Multi-Mode Microplate Reader (BioTek, Winooski, VT, USA).

### 4.3. Hoechst 33342 Staining Assay

Cells (5 × 10^5^) were seeded in 6-well plates per well and incubated overnight. Then cells were treated with 0–4 μM of TSE1 for 24 h, and stained with Hoechst 33342 (10 μg/mL) in the dark for 10 min. Cell apoptosis was observed using a Zeiss inverted fluorescence microscope in random microscopic fields.

### 4.4. Flow Cytometry Analysis for Apoptosis and Cell Cycle

Cells were treated with 0–4 μM of TSE1 for 24 h, and were collected by centrifugation (1500 rpm for 10 min) after digestion with trypsin. The apoptotic cells were determined by an Alexa Fluor 488 Annexin V/Dead Cell Apoptosis Kit (Invitrogen, Grand Island, NY, USA) according to the protocol. Briefly, cells were washed twice with ice cold phosphate buffer saline (PBS), and suspended in annexin-binding buffer with annexin V and propidium iodide (PI) solution. After staining for 15 min, cells were analyzed by a flow cytometry system (FACSCaliber system, BD Biosciences, San Jose, CA, USA), with excitation/emission wavelength at 488/530 nm. For cell cycle analysis, the cell pellet was suspended in 70% ethanol at −20 °C overnight, and washed twice with cold PBS. After incubation with 50 μL of RNase A (180 μg/mL) at 37 °C for 15 min, cells were stained with PI (50 μg/mL) for another 15 min. Flow cytometry analysis was performed with excitation wavelength at 535 nm and emission wavelength at 615 nm.

### 4.5. Wound Healing Assay

Cells were seeded into 6-well plates and allowed to reach about 80–90% confluence. The cell monolayer was scratched using a 200 μL pipet tip and was washed twice with PBS to get rid of debris. The serum-free RPMI 1640 medium containing TSE1 (0–4 μM) was added later on, and the cells were photographed under the light microscope (ZEISS, Heidelberg, Germany) at 0 and 24 h to measure the distance between the wounds.

### 4.6. Chicken Chorioallantoic Membrane (CAM) Assay

Specific-pathogen-free (SPF) fertile eggs (Charles River laboratories, Wilmington, MA, USA) were incubated and turned slowly by an automatic egg turner at 37.5 °C. At day 8, the CAM was exposed after opening a 1-cm diameter window in the egg shell. OVCAR-3 cells (1 × 10^6^) were suspended in a 1:4 (*v*/*v*) solution of serum-free medium and Matrigel, with or without 4 μM of TSE1. This cell suspension was implanted onto the CAM on the 10th day. Eight eggs were used for each group. After incubation for another 5 days, blood vessels were counted and photographed.

### 4.7. Enzyme Linked Immunosorbent Assay (ELISA)

OVCAR-3 cells were seeded at 1 × 10^4^ per well in 96-well plates, cultured overnight, and treated with 0–4 μM of TSE1 for 24 h. The VEGF in the cell culture media was determined using a human VEGF Duo-set ELISA kit (R&D, Minneapolis, MN, USA) according to the protocol.

### 4.8. Western Blot Assay

OVCAR-3 cells (1 × 10^6^) were incubated in 60-mm dishes overnight, then treated with 0–4 μM of TSE1 for 24 h. Afterwards, cells were lysed by mammalian protein extraction reagent supplemented with a cocktail of Halt protease and phosphatase inhibitor (Pierce, Rockford, IL, USA). The protein content was determined by a Pierce BCA protein assay kit, separated by sodium dodecyl sulfate-polyacrylamide gel electrophoresis (SDS-PAGE), and then immediately transferred onto nitrocellulose membranes. After blocking with 5% skim milk prepared in Tris-buffer saline plus 0.1% Tween 20 (TBST), the membranes were probed with primary and secondary antibodies successively. The antibodies were obtained from Santa Cruz Biotechnology Inc. (Mariposa, CA, USA) and Cell Signaling Technology Inc. (Danvers, MA, USA). Bands were visualized by SuperSignal West Dura Extended Duration Substrate (Thermo, Waltham, MA, USA) and analyzed using ImageJ (NIH, Bethesda, MD, USA).

### 4.9. Statistical Analysis

The data were presented as means ± standard deviations (SD) of at least three replicates. Statistical analyses were performed using the SAS system for windows V8 (SAS Institute Inc., Cary, NC, USA). Multiple comparisons were analyzed by one-way analysis of variance (ANOVA) followed with Student–Newman–Keuls (SNK) test. Significant statistical difference was set at *p* < 0.05.

## 5. Conclusions

In summary, this work demonstrated that TSE1 had more potent inhibitory effects on the platinum-resistant ovarian cancer OVCAR-3 and A2780/CP70 cells than cisplatin, and was less cytotoxic to normal ovarian IOSE-364 cells. TSE1 induced apoptosis of OVCAR-3 cells through both the intrinsic and extrinsic apoptotic pathways, strongly inhibited cell migration and attenuated angiogenesis in the CAM model. The Notch1/ATM/PTEN/Akt/mTOR/HIF-1α axis plays a key role in the anticancer activity of TSE1. Combination treatment of TSE1 with the Notch1 signaling inhibitor DAPT, or Akt signaling inhibitor wortmannin, showed stronger inhibition towards HIF-1α protein expression compared with single compound-treatment. TSE1 is a potential candidate for improving platinum-resistant ovarian cancer, and *C. sinensis* seeds are a promising source of this active saponin.

## Data Availability

Data available in a publicly accessible repository.

## References

[1] Mirza-Aghazadeh-Attari M., Ostadian C., Saei A.A., Mihanfar A., Darband S.G., Sadighparvar S., Kaviani M., Kafil H.S., Yousefi B., Majidinia M. (2019). DNA damage response and repair in ovarian cancer: Potential targets for therapeutic strategies. DNA Repair.

[2] Lee A.W., Navajas E.E., Liu L.H. (2019). Clear differences in ovarian cancer incidence and trends by ethnicity among Asian Americans. Cancer Epidemiol..

[3] Chornokur G., Amankwah E.K., Schildkraut J.M., Phelan C.M. (2013). Global ovarian cancer health disparities. Gynecol. Oncol..

[4] Shen F., Zong Z.H., Liu Y., Chen S., Sheng X.J., Zhao Y. (2019). CEMIP promotes ovarian cancer development and progression via the PI3K/AKT signaling pathway. Biomed. Pharmacother..

[5] Mullen M.M., Kuroki L.M., Thaker P.H. (2019). Novel treatment options in platinum-sensitive recurrent ovarian cancer: A review. Gynecol. Oncol..

[6] Qiu Y.M., Liu P.S., Ma X.M., Ma X.X., Zhu L.H., Lin Y.K., You Y.J., Yu W.H., Ma D.P., Sun C.Y. (2019). TRIM50 acts as a novel Src suppressor and inhibits ovarian cancer progression. BBA Mol. Cell Res..

[7] Samper K.G., Marker S.C., Bayon P., MacMillan S.N., Keresztes I., Palacios O., Wilson J.J. (2017). Anticancer activity of hydroxy- and sulfonamide-azobenzene platinum(II) complexes in cisplatin-resistant ovarian cancer cells. J. Inorg. Biochem..

[8] Simpkins F., Jang K., Yoon H., Hew K.E., Kim M., Azzam D.J., Sun J., Zhao D.K., Ince T.A., Liu W.B. (2018). Dual Src and MEK Inhibition Decreases Ovarian Cancer Growth and Targets Tumor Initiating Stem-Like Cells. Clin. Cancer Res..

[9] Lim B., Greer Y., Lipkowitz S., Takebe N. (2019). Novel apoptosis-inducing agents for the treatment of cancer, a new arsenal in the toolbox. Cancers.

[10] Yang Y.N., Li S., Sun Y.T., Zhang D., Zhao Z.Y., Liu L. (2019). Reversing platinum resistance in ovarian cancer multicellular spheroids by targeting Bcl-2. Oncotargets Ther..

[11] Hassan H.A., Salem M.L., Gouida M.S., El-Azab K.M. (2018). Comparative expression of caspases and annexin V in benign and malignant ovarian tumors. J. Cancer Res. Ther..

[12] Yan X.Y., Zhong X.R., Yu S.H., Zhang L.C., Liu Y.N., Zhang Y., Sun L.K., Su J. (2019). p62 aggregates mediated Caspase 8 activation is responsible for progression of ovarian cancer. J. Cell Mol. Med..

[13] Kim M., Hernandez L., Annunziata C.M. (2016). Caspase 8 expression may determine the survival of women with ovarian cancer. Cell Death Dis..

[14] Schmitt J., Matei D. (2012). Targeting angiogenesis in ovarian cancer. Cancer Treat. Rev..

[15] Chen X.H., Mangala L.S., Mooberry L., Bayraktar E., Dasari S.K., Ma S.L., Ivan C., Court K.A., Rodriguez-Aguayo C., Bayraktar R. (2019). Identifying and targeting angiogenesis-related microRNAs in ovarian cancer. Oncogene.

[16] Wang H.H., Xu T.E., Zheng L.F., Li G.L. (2018). Angiogenesis inhibitors for the treatment of ovarian cancer: An updated systematic review and meta-analysis of randomized controlled trials. Int. J. Gynecol. Cancer.

[17] Lindgren A., Anttila M., Rautiainen S., Arponen O., Hamalainen K., Kononen M., Vanninen R., Sallinen H. (2019). Dynamic contrast-enhanced perfusion parameters in ovarian cancer: Good accuracy in identifying high HIF-1 alpha expression. PLoS ONE.

[18] Zhao T., Zhao C.Y., Zhou Y.T., Zheng J., Gao S.J., Lu Y. (2017). HIF-1 alpha binding to AEG-1 promoter induced upregulated AEG-1 expression associated with metastasis in ovarian cancer. Cancer Med..

[19] Long F.Y., Liu W.X., Jia P., Wang H.F., Jiang G., Wang T. (2018). HIF-1 alpha-induced autophagy contributes to cisplatin resistance in ovarian cancer cells. Pharmazie.

[20] Ai Z.H., Lu Y., Qiu S.B., Fan Z. (2016). Overcoming cisplatin resistance of ovarian cancer cells by targeting HIF-1-regulated cancer metabolism. Cancer Lett..

[21] Ding Y., Ding C.Y., Ye N., Liu Z.Q., Wold E.A., Chen H.Y., Wild C., Shen Q., Zhou J. (2016). Discovery and development of natural product oridonin-inspired anticancer agents. Eur. J. Med. Chem..

[22] Jeepipallia S.P.K., Du B., Sabitaliyevich U.Y., Xu B. (2020). New insights into potential nutritional effects of dietary saponins in protecting against the development of obesity. Food Chem..

[23] Xu X.H., Li T., Fong C.M.V., Chen X.P., Chen X.J., Wang Y.T., Huang M.Q., Lu J.J. (2016). Saponins from chinese medicines as anticancer agents. Molecules.

[24] Guo N., Tong T.T., Ren N., Tu Y.Y., Li B. (2018). Saponins from seeds of Genus *Camellia*: Phytochemistry and bioactivity. Phytochemistry.

[25] Li N., Ma Z.J., Chu Y., Wang Y., Li X. (2013). Phytochemical analysis of the triterpenoids with cytotoxicity and QR inducing properties from the total tea seed saponin of *Camellia sinensis*. Fitoterapia.

[26] Jia L.Y., Xia H.L., Chen Z.D., Compton C., Bucur H., Sawant D.A., Rankin G.O., Li B., Tu Y.Y., Chen Y.C. (2018). Anti-proliferation effect of theasaponin E-1 on the ALDH-positive ovarian cancer stem-like cells. Molecules.

[27] Zhao P.X., Li M.S., Chen Y., He C.C., Zhang X.J., Fan T., Yang T., Lu Y., Lee R.J., Ma X. (2019). Selenium-doped calcium carbonate nanoparticles loaded with cisplatin enhance efficiency and reduce side effects. Int. J. Pharm..

[28] Shirjang S., Mansoori B., Asghari S., Duijf P.H.G., Mohammadi A., Gjerstorff M., Baradaran B. (2019). MicroRNAs in cancer cell death pathways: Apoptosis and necroptosis. Free Radic. Biol. Med..

[29] Derakhshan A., Chen Z., Van Waes C. (2017). Therapeutic small molecules target inhibitor of apoptosis proteins in cancers with deregulation of extrinsic and intrinsic cell death pathways. Clin. Cancer Res..

[30] Yang S., Mao Y.J., Zhang H.J., Xu Y., An J., Huang Z.W. (2019). The chemical biology of apoptosis: Revisited after 17 years. Eur. J. Med. Chem..

[31] Seo J., Kim M.W., Bae K.H., Lee S.C., Song J., Lee E.W. (2019). The roles of ubiquitination in extrinsic cell death pathways and its implications for therapeutics. Biochem. Pharmacol..

[32] Sachan R., Kundu A., Jeon Y., Choi W.S., Yoon K., Kim I.S., Kwak J.H., Kim H.S. (2018). Afrocyclamin A, a triterpene saponin, induces apoptosis and autophagic cell death via the PI3K/Akt/mTOR pathway in human prostate cancer cells. Phytomedicine.

[33] Zong J.F., Wang D.X., Jiao W.T., Zhang L., Bao G.H., Ho C.T., Hou R.Y., Wan X.C. (2016). Oleiferasaponin C-6 from the seeds of *Camellia oleifera* Abel.: A novel compound inhibits proliferation through inducing cell-cycle arrest and apoptosis on human cancer cell lines in vitro. RSC Adv..

[34] Mo S.S., Xiong H., Shu G.W., Yang X.Z., Wang J.X., Zheng C.Y., Xiong W., Mei Z.N. (2013). Phaseoloideside E, a novel natural triterpenoid saponin identified from entada phaseoloides, induces apoptosis in Ec-109 esophageal cancer cells through reactive oxygen species generation. J. Pharmacol. Sci..

[35] Stucki M. (2009). Histone H2A.X Tyr142 phosphorylation: A novel sWItCH for apoptosis?. DNA Repair.

[36] Roos W.P., Kaina B. (2013). DNA damage-induced cell death: From specific DNA lesions to the DNA damage response and apoptosis. Cancer Lett..

[37] Wang Z., Chen Q., Li B., Xie J.M., Yang X.D., Zhao K., Wu Y., Ye Z.Y., Chen Z.R., Qin Z.H. (2018). Escin-induced DNA damage promotes escin-induced apoptosis in human colorectal cancer cells via p62 regulation of the ATM/gamma H2AX pathway. Acta Pharmacol. Sin..

[38] Iness A.N., Litovchick L. (2018). MuvB: A key to cell cycle control in ovarian cancer. Front. Oncol..

[39] Shin S.S., Hwang B., Muhammad K., Gho Y., Song J.H., Kim W.J., Kim G., Moon S.K. (2019). Nimbolide represses the proliferation, migration, and invasion of bladder carcinoma cells via Chk2-mediated G2/M phase cell cycle arrest, altered signaling pathways, and reduced transcription factors-associated MMP-9 expression. Evid. Based Complement. Altern..

[40] Gogineni V.R., Nalla A.K., Gupta R., Dinh D.H., Klopfenstein J.D., Rao J.S. (2011). Chk2-mediated G2/M cell cycle arrest maintains radiation resistance in malignant meningioma cells. Cancer Lett..

[41] Garrido M.P., Torres I., Vega M., Romero C. (2019). Angiogenesis in Gynecological Cancers: Role of neurotrophins. Front. Oncol..

[42] Caporarello N., Lupo G., Olivieri M., Cristaldi M., Cambria M.T., Salmeri M., Anfuso C.D. (2017). Classical VEGF, Notch and Ang signalling in cancer angiogenesis, alternative approaches and future directions (Review). Mol. Med. Rep..

[43] Han Y.Q., Pan L.Y., Ran S., Song Y., Sun F.F., Wang Y.Z., Hong Y. (2019). *Rhizoma Paridis* saponins ameliorates hepatic fibrosis in rats by downregulating expression of angiogenesis-associated growth factors. Mol. Med. Rep..

[44] Hui Z., Sha D.J., Wang S.L., Li C.S., Qian J., Wang J.Q., Zhao Y., Zhang J.H., Cheng H.Y., Yang H. (2017). Panaxatriol saponins promotes angiogenesis and enhances cerebral perfusion after ischemic stroke in rats. BMC Complement. Altern. Med..

[45] Bian X.B., Zhao Y., Guo X., Zhang L.X., Li P.Y., Fu T.H., Wang W.D., Yin Y.X., Chen G.L., Liu J.P. (2017). Chiisanoside, a triterpenoid saponin, exhibits anti-tumor activity by promoting apoptosis and inhibiting angiogenesis. RSC Adv..

[46] Rajasekar J., Perumal M.K., Vallikannan B. (2019). A critical review on anti-angiogenic property of phytochemicals. J. Nutr. Biochem..

[47] Duan P., Fan L.L., Gao Q.S., Silwal B.M., Ren M.L., Shen Y., Qu W.L. (2017). Targeted therapy of ovarian cancer with angiogenesis inhibitors. Curr. Drug Targets.

[48] Redfern A., Agarwal V., Thompson E.W. (2019). Hypoxia as a signal for prison breakout in cancer. Curr. Opin. Clin. Nutr..

[49] Courtnay R., Ngo D.C., Malik N., Ververis K., Tortorella S.M., Karagiannis T.C. (2015). Cancer metabolism and the Warburg effect: The role of HIF-1 and PI3K. Mol. Biol. Rep..

[50] Mi C.L., Ma J., Shi H., Li J., Wang F., Lee J.J., Jin X.J. (2014). 4′,6-Dihydroxy-4-methoxyisoaurone inhibits the HIF-1 alpha pathway through inhibition of Akt/mTOR/p70S6K/4E-BP1 phosphorylation. J. Pharmacol. Sci..

[51] Ataie-Kachoie P., Pourgholami M.H., Bahrami-B F., Badar S., Morris D.L. (2015). Minocycline attenuates hypoxia-inducible factor-1 alpha expression correlated with modulation of p53 and AKT/mTOR/p70S6K/4E-BP1 pathway in ovarian cancer: In vitro and in vivo studies. Am. J. Cancer Res..

[52] Huang J.L., Gao L.K., Li B.S., Liu C., Hong S.S., Min L., Hong L. (2019). Knockdown of hypoxia-inducible factor 1 alpha (HIF-1 alpha) promotes autophagy and inhibits phosphatidylinositol 3-Kinase (PI3K)/AKT/mammalian target of rapamycin (mTOR) signaling pathway in ovarian cancer cells. Med. Sci. Monit..

[53] Zhang M.F., Hagan C.T., Min Y.Z., Foley H., Tian X., Yang F.F., Mi Y., Au K.M., Medik Y., Roche K. (2018). Nanoparticle co-delivery of wortmannin and cisplatin synergistically enhances chemoradiotherapy and reverses platinum resistance in ovarian cancer models. Biomaterials.

[54] Ortiz T., Burguillos M.A., Lopez-Lluch G., Navas P., Herrador M., Gonzalez I., Pinero J. (2008). Enhanced induction of apoptosis in a radio-resistant bladder tumor cell line by combined treatments with X-rays and wortmannin. Radiat. Environ. Biophys..

[55] Kantidze O.L., Velichko A.K., Luzhin A.V., Petrova N.V., Razin S.V. (2018). Synthetically lethal interactions of ATM, ATR, and DNA-PKcs. Trends Cancer.

[56] Abdel-Fatah T.M., Arora A., Moseley P., Coveney C., Perry C., Johnson K., Kent C., Ball G., Chan S., Madhusudan S. (2014). ATM, ATR and DNA-PKcs expressions correlate to adverse clinical outcomes in epithelial ovarian cancers. BBA Clin..

[57] Yin S., Wang P., Yang L.N., Liu Y., Wang Y., Liu M.M., Qi Z.H., Meng J., Shi T.Y., Yang G. (2016). Wip1 suppresses ovarian cancer metastasis through the ATM/AKT/Snail mediated signaling. Oncotarget.

[58] Liu H.Y., Zhang Y.Y., Zhu B.L., Feng F.Z., Zhang H.T., Yan H., Zhou B. (2019). MiR-203a-3p regulates the biological behaviors of ovarian cancer cells through mediating the Akt/GSK-3 beta/Snail signaling pathway by targeting ATM. J. Ovarian Res..

[59] Ali R., Alabdullah M., Miligy I., Normatova M., Babaei-Jadidi R., Nateri A.S., Rakha E.A., Madhusudan S. (2019). ATM regulated PTEN degradation is XIAP E3 ubiquitin ligase mediated in p85alpha deficient cancer cells and influence platinum sensitivity. Cells.

[60] Chen J., Bai M., Ning C., Xie B., Zhang J., Liao H., Xiong J., Tao X., Yan D., Xi X. (2016). Gankyrin facilitates follicle-stimulating hormone-driven ovarian cancer cell proliferation through the PI3K/AKT/HIF-1 alpha/cyclin D1 pathway. Oncogene.

[61] Ma Y.C., Su N., Shi X.J., Zhao W., Ke Y., Zi X.L., Zhao N.M., Qin Y.H., Zhao H.W., Liu H.M. (2015). Jaridonin-induced G2/M phase arrest in human esophageal cancer cells is caused by reactive oxygen species-dependent Cdc2-tyr15 phosphorylation via ATM-Chk1/2-Cdc25C pathway. Toxicol. Appl. Pharm..

[62] Aster J.C., Pear W.S., Blacklow S.C. (2017). The varied roles of Notch in cancer. Annu. Rev. Pathol. Mech..

[63] Groeneweg J.W., Foster R., Growdon W.B., Verheijen R.H.M., Rueda B.R. (2014). Notch signaling in serous ovarian cancer. J. Ovarian Res..

[64] Rose S.L., Kunnimalaiyaan M., Drenzek J., Seiler N. (2010). Notch 1 signaling is active in ovarian cancer. Gynecol. Oncol..

[65] Huang J., Hu W., Hu L.M., Previs R.A., Dalton H.J., Yang X.Y., Sun Y.J., McGuire M., Rupaimoole R., Nagaraja A.S. (2016). Dll4 inhibition plus aflibercept markedly reduces ovarian tumor growth. Mol. Cancer Ther..

[66] Yang J., Xing H., Lu D.H., Wang J., Li B.S., Tang J.M., Gu F.Q., Hong L. (2019). Role of Jagged1/STAT3 signalling in platinum-resistant ovarian cancer. J. Cell Mol. Med..

[67] Vermezovic J., Adamowicz M., Santarpia L., Rustighi A., Forcato M., Lucano C., Massimiliano L., Costanzo V., Bicciato S., Del Sal G. (2015). Notch is a direct negative regulator of the DNA-damage response. Nat. Struct. Mol. Biol..

[68] Kim T.H., Park J.H., Woo J.S. (2019). Resveratrol induces cell death through ROS-dependent downregulation of Notch1/PTEN/Akt signaling in ovarian cancer cells. Mol. Med. Rep..

[69] Shen F.H., Xiong Z.W., Kong J.M., Wang L., Cheng Y.S., Jin J., Huang Z.Y. (2019). Triptolide impairs thioredoxin system by suppressing Notch1-mediated PTEN/Akt/Txnip signaling in hepatocytes. Toxicol. Lett..

[70] Poddar S., Kesharwani D., Datta M. (2019). miR-449a regulates insulin signalling by targeting the Notch ligand, Jag1 in skeletal muscle cells. Cell Commun. Signal..

[71] Li S.R., Ren B., Shi Y., Gao H., Wang J.W., Xin Y., Huang B., Liao S.C., Yang Y.P., Xu Z.X. (2019). Notch1 inhibition enhances DNA damage induced by cisplatin in cervical cancer. Exp. Cell Res..

[72] Chen X.X., Gong L.H., Ou R.Y., Zheng Z.Z., Chen J.Y., Xie F.F., Huang X.X., Qiu J., Zhang W.J., Jiang Q.W. (2016). Sequential combination therapy of ovarian cancer with cisplatin and gamma-secretase inhibitor MK-0752. Gynecol. Oncol..

[73] Böttger S., Melzig M.F. (2013). The influence of saponins on cell membrane cholesterol. Bioorg. Med. Chem..

[74] Wu X., Jia L., Wu J., Liu Y., Kang H., Liu X., Li P., He P., Tu Y., Li B. (2019). Simultaneous determination and quantification of triterpene saponins from *Camellia sinensis* seeds using UPLC-PDA-QTOF-MS/MS. Molecules.

